# Supplementary material for: diffBUM-HMM: a robust statistical modeling approach for detecting RNA flexibility changes in high-throughput structure probing data
Source: Genome Biol. 2021 May 27;22:165. doi: 10.1186/s13059-021-02379-y (PMC8157727; doi:10.1186/s13059-021-02379-y)
Supplement: Supplementary file 1 — Additional file 1 Figure S1–S7. Figure S1: Optimization of the diffBUM-HMM transition matrix: boxplots of prediction mismatch value over transition matrix perturbations for the 35S and Xist molecules. Figure S2: Enriched RBP binding motif search amongst the DRNs/DRRs detected by diffBUM-HMM and deltaSHAPE within the Xist molecule. Figure S3: Distribution of log drop-off rate ratios (LDRs) and P values for the 35S data. Figure S4: Distribution of log mutation rate ratios (LMRs) and P values for the Xist data. Figure S5: Distribution of log drop-off rate ratios (LDRs) and P values for the rRNA control datasets. Figure S6: Interpreting the output of diffBUM-HMM: diffBUM-HMM pipeline output in contrast with drop-off rates and P values. Figure S7: Discrepancies between deltaSHAPE and diffBUM-HMM explained by noise in the data: A comparison between the individual replicates of deltaSHAPE analyses for the 35S molecule. [file 13059_2021_2379_MOESM1_ESM.pdf]

Supplementary material for

diffBUM-HMM: a robust statistical modelling approach for detecting RNA flexibility changes in high-throughput structure probing data

Paolo Marangio[2,3,*], Ka Ying Toby Law[1,*], Guido Sanguinetti[1,2,3,†] and Sander Granneman[1,†]

[1]Centre for Synthetic and Systems Biology, The University of Edinburgh, Edinburgh, UK
[2]School of Informatics, The University of Edinburgh, Edinburgh, UK
[3]SISSA, Data Science Excellence Department Initiative, Trieste, Italy
[*]These authors contributed equally
[†]Corresponding authors: Sander Granneman, e-mail: sander.granneman@ed.ac.uk and Guido Sanguinetti, e-mail: gsanguin@sissa.it

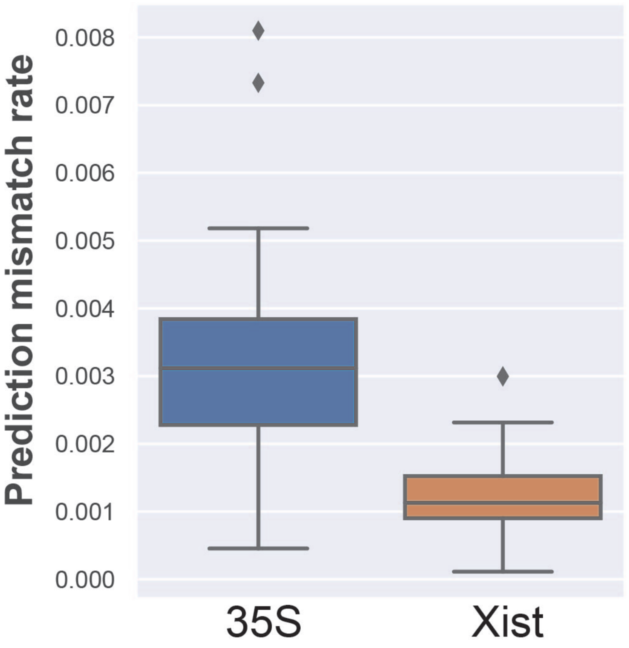

Fig. S1: **Optimization of diffBUM-HMM transition matrix.** Boxplot of prediction mismatch value over 52 transition matrix perturbations for 35S molecule and 61 for Xist molecule. A conservative approach was used in order to adapt the transition probabilities, such that the original values would be respected. Perturbation tests were conducted in order to determine whether the adapted values were indeed optimal. Random Gaussian noise with mean 0 and standard deviation 0.01 was added to the first 3 transition probabilities for each state, while the last transition probability was set such that the 4 values would add up to 1. The resulting posterior probabilities for the differential states (i.e. *hidden state* values 2 and 3) at each position generated when diffBUM-HMM with the noisy transition matrix was applied to the data were then compared against the values outputted with the original, noise-free matrix. A prediction mismatch score can then be computed by dividing the number of incorrect predictions by the number of correct predictions over the entire molecule length, indicating the prediction mismatch associated with an individual noisy configuration of the transition matrix. The prediction mismatch for the 35S pre-rRNA and Xist molecules averaged over more than 50 different, noisy configurations of the transition matrix was smaller than 0.5%. This suggests that the transition matrix configuration used to analyse these datasets was optimal.

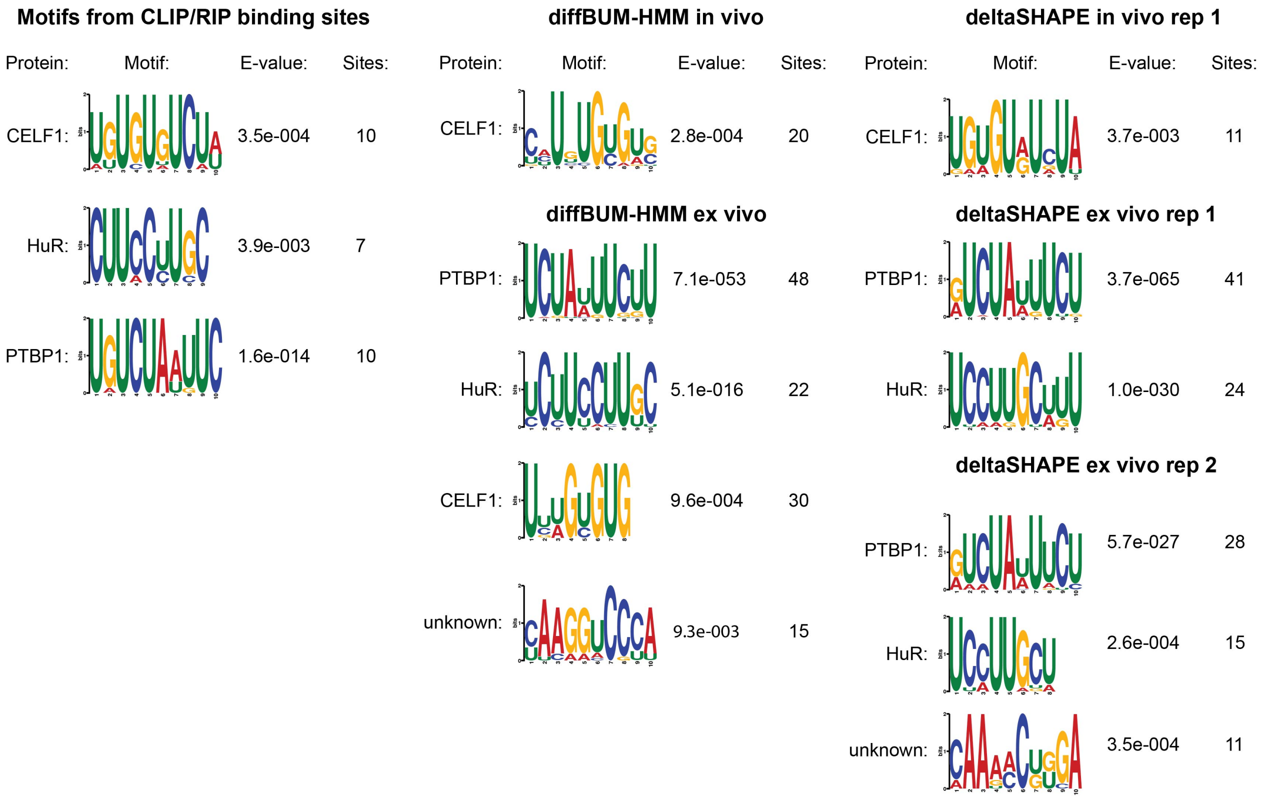

Fig. S2: **DiffBUM-HMM DRNs in the Xist molecule are enriched in protein-binding sites**. To detect enriched RBP binding motifs in Xist, DRNs in the *ex vivo* data that were located within a window of 5 nucleotides were grouped into a single interval, each of which was subsequently extended to 30 nucleotides. To identify enriched sequence motifs, these intervals were analysed using the MEME tool suite as described in the Methods section. The left panel shows the sequence motifs that were detected in the protein-binding sites detected by CLIP/RIP. The middle panel shows the detected enriched motifs surrounding diffBUM-HMM DRNs and the right panel shows those for the deltaSHAPE analysis of individual replicates. The E-value is the expectation of observing a particular motif by chance. Only motifs with an E-value ≤ 0.05 are shown.On the left of each motif the name of the protein that is predicted to bind these sequences is indicated. Unknown indicates that it is unclear what protein is binding this motif (if any).

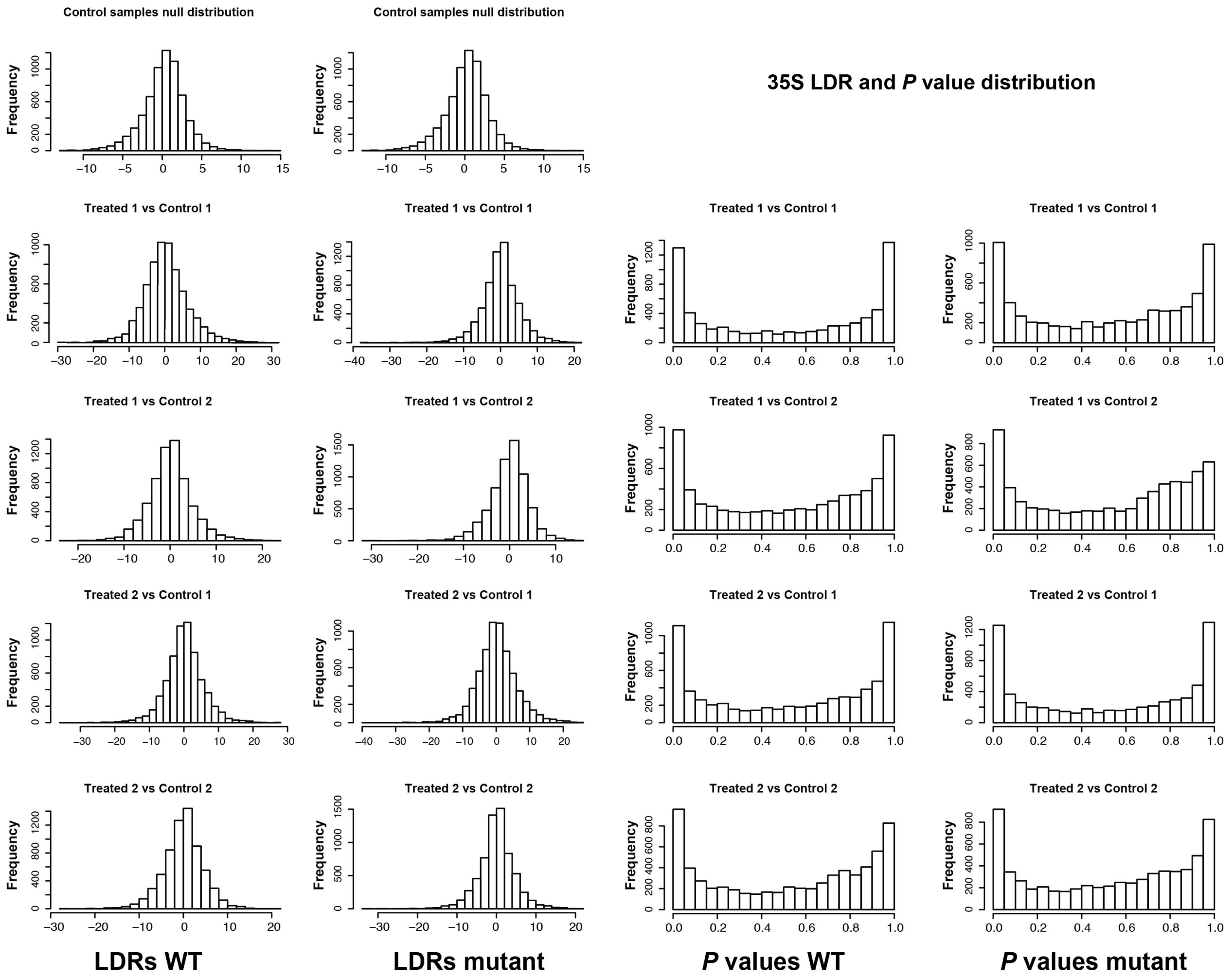

Fig. S3: **Distribution of log drop-off rate ratios (LDRs) and P values for the 35S data.** 'Treated' and 'Control' indicate the individual replicates of the SHAPE-modified and DMSO-treated samples, respectively. 'WT' indicates 35S pre-rRNA affinity purified using epitope-tagged wild-type Mrd1 as bait, while mutant indicates that the Mrd1 $\Delta 5$ variant has been used as bait.

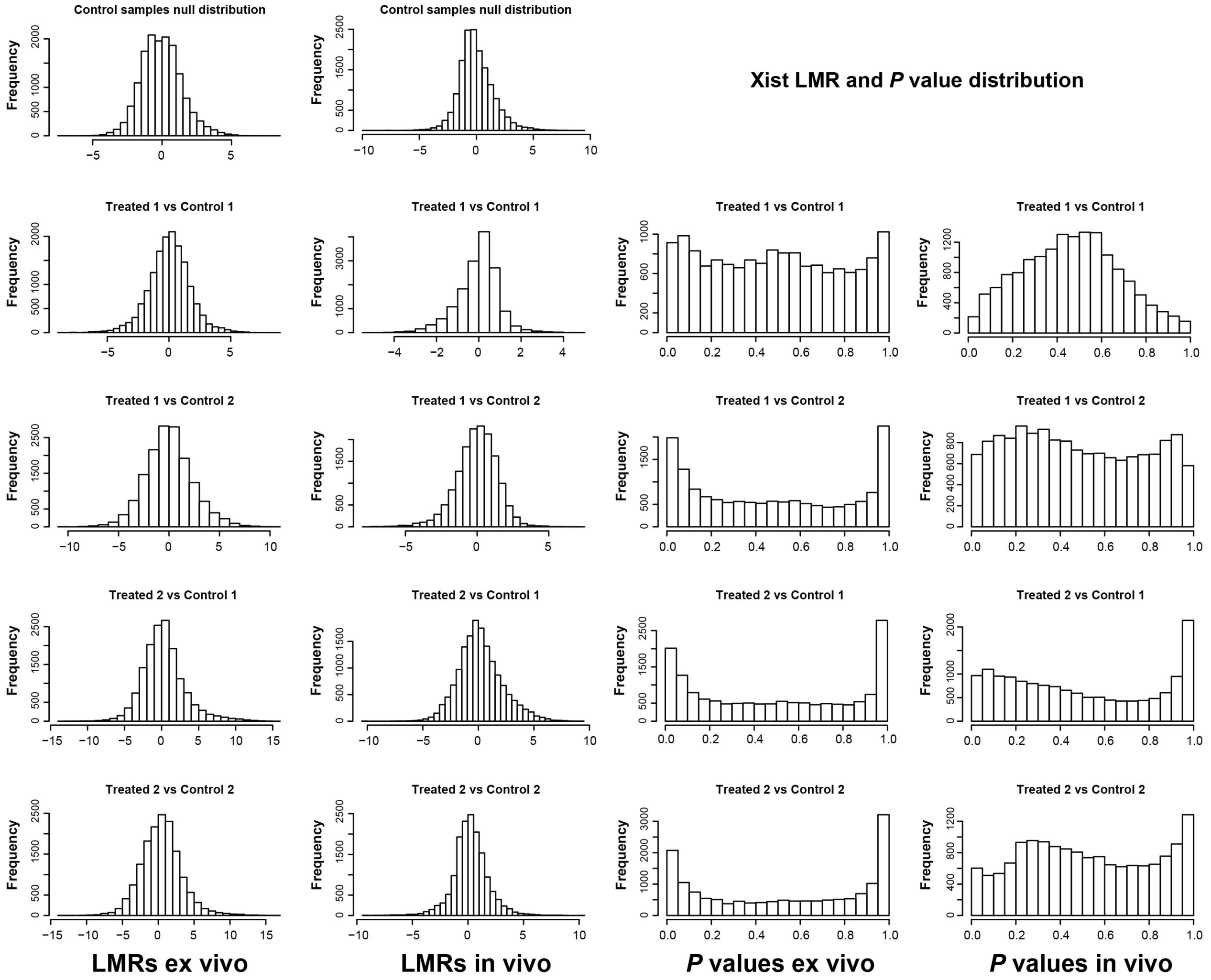

Fig. S4: **Distribution of log mutation rate ratios (LMRs) and *P* values for the Xist data.** 'Treated' and 'Control' indicate the individual replicates of the SHAPE-modified and DMSO-treated samples, respectively.

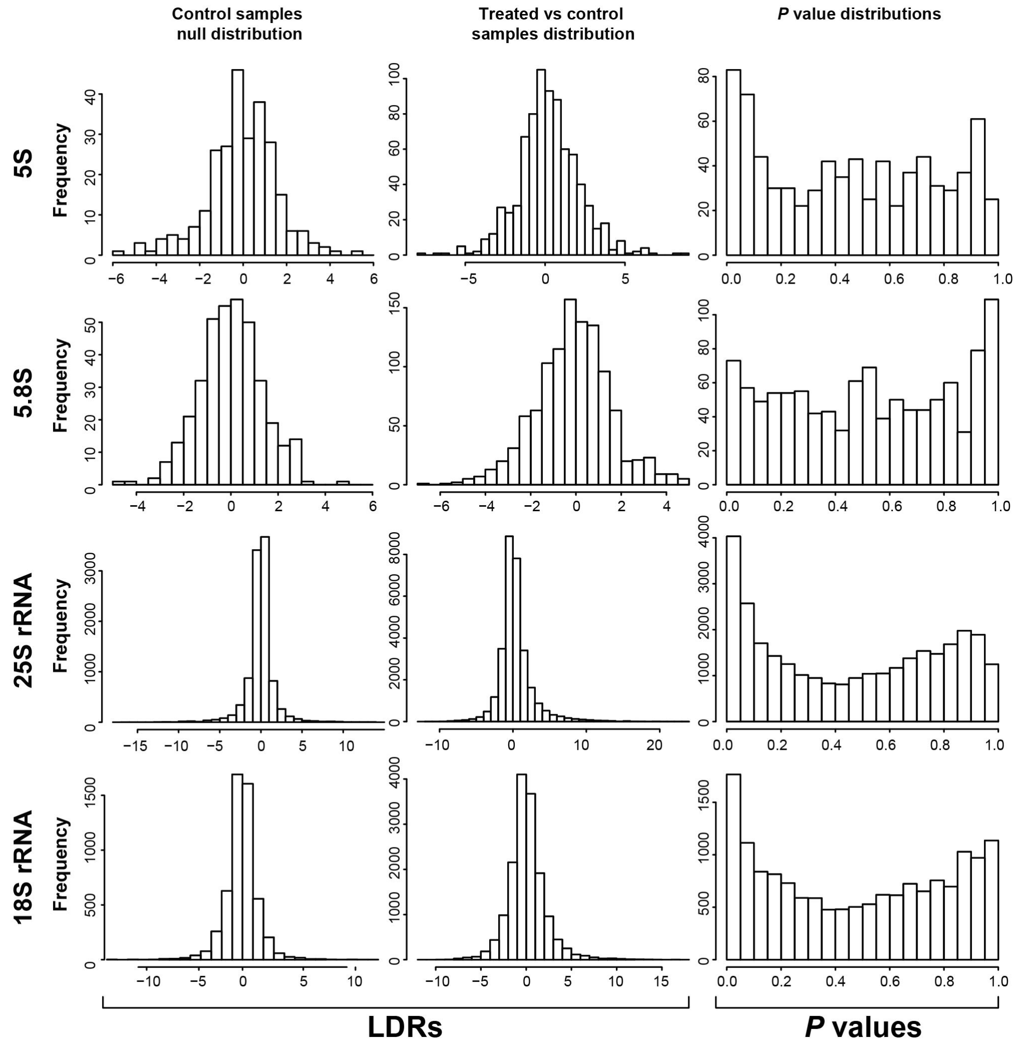

Fig. S5: **Distribution of log drop-off rate ratios (LDRs) and *P* values for the rRNA control datasets.** Since the replicates of the control and treated samples showed a very similar distribution, the data from the different replicates were merged into a single plot. 'Treated' and 'Control' indicate the samples with and without DMS treatment, respectively.

A

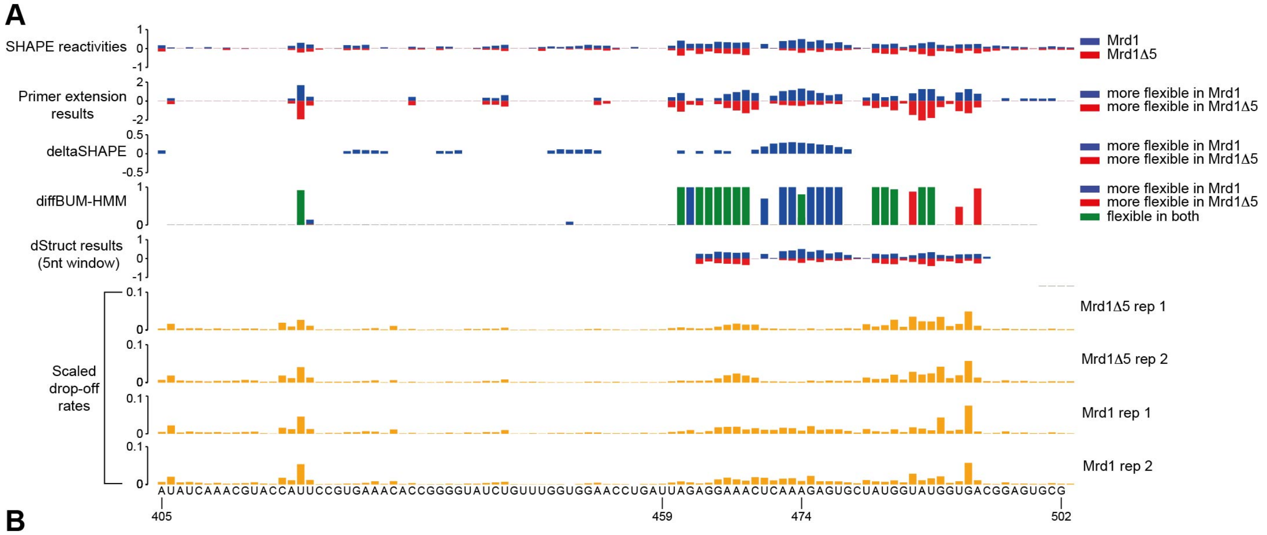

B

nt		459	460	461	462	463	464	465	466	467	468	469	470	471	472	473	474
Mrd1 Δ5		0.91	0.32	0.019	0.24	0.026	0.130	0.088	0.091	0.170	0.040	0.86	0.63	0.91	0.42	0.23	0.15
		0.84	0.21	0.020	0.37	0.088	0.350	0.110	0.100	0.043	0.038	0.68	0.54	0.85	0.48	0.30	0.18
		0.97	0.56	0.016	0.28	0.011	0.068	0.039	0.032	0.065	0.014	0.91	0.79	0.96	0.43	0.40	0.11
		0.93	0.41	0.018	0.44	0.040	0.240	0.049	0.038	0.014	0.014	0.73	0.72	0.93	0.51	0.50	0.13
Mrd1		0.94	0.31	0.011	0.063	0.020	0.038	0.019	0.037	0.110	0.036	0.88	0.220	0.76	0.016	0.010	0.006
		0.88	0.19	0.012	0.110	0.085	0.140	0.023	0.043	0.021	0.035	0.68	0.150	0.59	0.020	0.013	0.008
		0.89	0.31	0.015	0.230	0.045	0.120	0.048	0.063	0.230	0.073	0.80	0.130	0.72	0.031	0.017	0.012
		0.82	0.21	0.016	0.350	0.140	0.330	0.057	0.070	0.065	0.070	0.55	0.095	0.56	0.039	0.021	0.014

Fig. S6: **diffBUM-HMM output in contrast with drop-off rates for 35S pre-rRNA region 405-502, and *P* values for region 459-474.** (**A**) Scaled drop-off rates (DORs) for each of the variants Mrd1Δ5 mutant and WT-Mrd1 provide an additional indicator of differential reactivity, alongside the diffBUM-HMM output for the region. Positive deltaSHAPE signals (blue) indicate regions that are more flexible in the strain expressing the wild-type Mrd1 protein. Negative deltaSHAPE signals (red) indicate regions that are more flexible in the MrdΔ5 mutant. Regions that diffBUM-HMM called modified or flexible in both pre-rRNA samples are indicated in green. (**B**) diffBUM-HMM predictions correspond with *P* values calculated for each treatment vs. control comparison for the two sets of 35S pre-rRNA variants. Positions with notable predictions are highlighted in red.

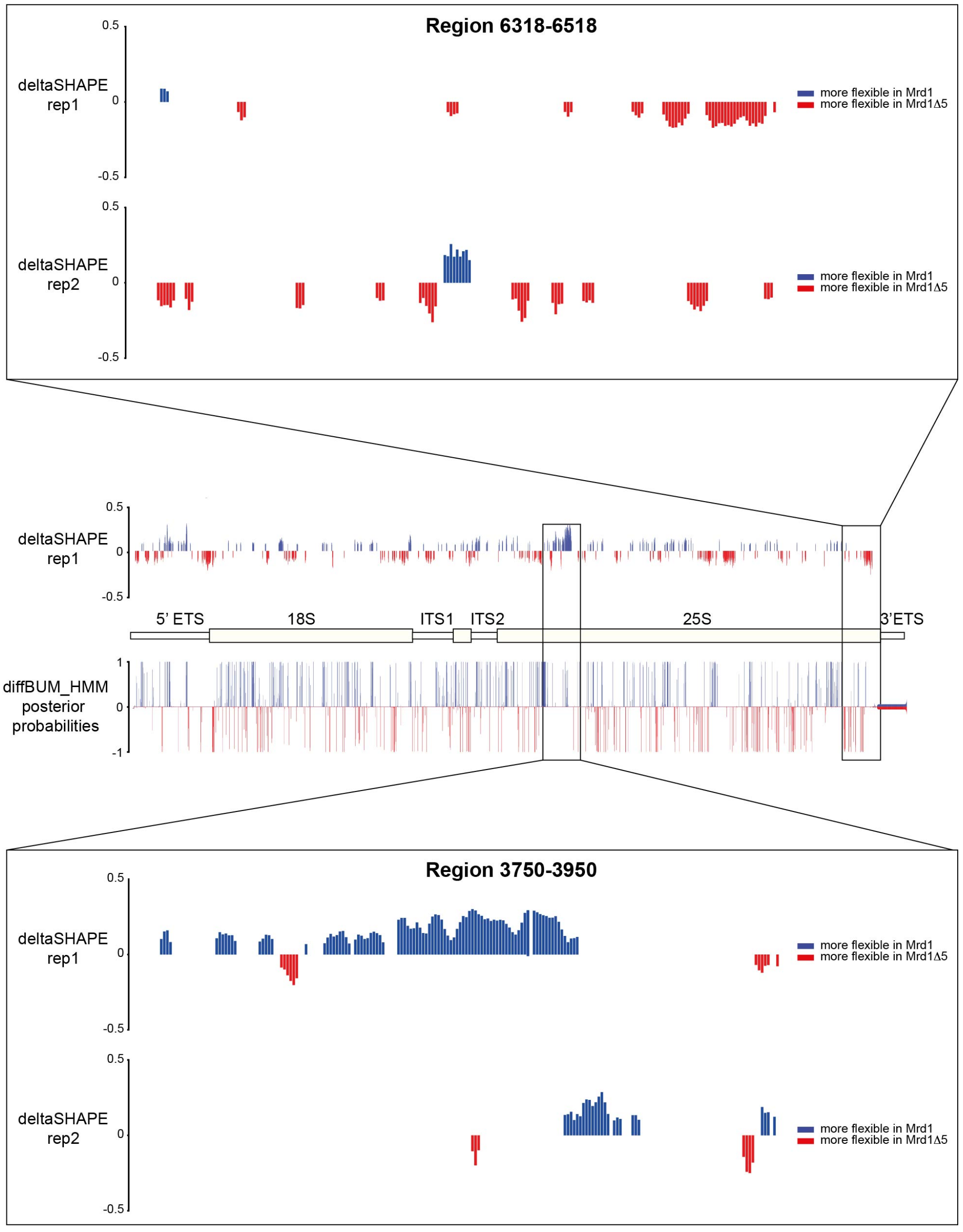

Fig. S7: **Discrepancies between deltaSHAPE and diffBUM-HMM results can be explained by noise in the data.** Comparison of deltaSHAPE analysis of the individual replicates of the 35S ChemModSeq data for the regions 3750-3950 and 6318-6518 in the 25S coding sequence of the 35S pre-rRNA. The rectangular boxes indicate the regions for which we show the deltaSHAPE results for the individual replicates. Positive deltaSHAPE signals (blue) indicate regions that are more flexible in the strain expressing the wild-type Mrd1 protein. Negative deltaSHAPE signals (red) indicate regions that are more flexible in the MrdΔ5 mutant.
